# Phylograd: fast column-specific calculation of substitution model gradients

**DOI:** 10.1186/s12859-025-06353-4

**Published:** 2025-12-23

**Authors:** Benjamin Lieser, Georgy Belousov, Johannes Söding

**Affiliations:** 1 Quantitative and Computational Biology, Max Planck Institute for Multidisciplinary Sciences, Am Faßberg 11, Göttingen, 37077 Germany; 2 International Max-Planck Research School for Genome Sciences, University of Göttingen, Göttingen, 37077 Germany; 3 Campus-Institut Data Science (CIDAS), University of Göttingen, Göttingen, 37077 Germany

**Keywords:** Phylogeny, Reverse-mode differentiation, Felsenstein, Site-specific models

## Abstract

**Background:**

Most popular tools for reconstructing phylogenetic trees from multiple sequence alignments use a model of molecular evolution in which a single substitution matrix or a small set of fixed matrices are shared between all columns. Models with column-specific rate matrices can in principle be fit by automatic differentiation methods, but in practice the heavy computational burden associated with computing the gradients of the many matrix exponentials has hindered exploration of such models.

**Implementation:**

Here, we present a highly efficient approach for reverse-mode differentiation of the log likelihood computed with Felsenstein’s algorithm under any time-reversible substitution model. PhyloGrad is implemented in Rust and has Python bindings to easily combine it with automatic differentiation tools.

**Results:**

Depending on the tree size, PhyloGrad is 30-100 times faster than automatic differentiation in Pytorch and uses 10-100 times less memory. Even in the task of fitting one global model it is still at least 10 times faster than IQ-TREE3. PhyloGrad accelerates current model optimizations and enables the field to easily explore and implement novel site-specific models.

**Supplementary Information:**

The online version contains supplementary material available at 10.1186/s12859-025-06353-4.

## Background

The process of amino acid or nucleotide substitution in a Multiple Sequence Alignment (MSA) is commonly modeled by a continuous-time Markov chain (CTMC). A CTMC is specified by a rate matrix $Q \in \mathbb {R}^{n \times n}$, where *n* is the number of different residues (amino acids or nucleotides), so typically 20 or 4. Given a phylogenetic tree of the sequences and the rate matrix, Felsenstein’s algorithm [1] can be used to calculate the likelihood of the MSA under this model.

Due to different selection pressures acting on different columns of the MSA, the process of residue substitution is different in each column. However, most of the models implemented in IQ-TREE3 [2] and RAxML [3] use a single parameter-less model like LG [4] or WAG [5], and add a rate model to account for differing overall mutation rates between columns. The models C10, C20, ..., C60 [6], inspired by CAT [7], are a popular choice to account for site-dependent selection pressures. They contain 10 to 60 amino acid profiles trained on a large amounts of MSAs. Except for the optional weights of mixture components, there are no free parameters.

For phylogenetic analysis on a larger alignment, it would be beneficial to fit more parameters. Even in the setting of Cxx models, the work of Baños et al. [8] points out that accuracy of these models can be improved by refitting the substitution matrices. In order to fit substitution matrices, an efficient way to compute their gradients is required. Currently the fitting on the input MSAs is discouraged because of the substantial runtimes involved.

Fast gradients also enable Bayesian estimates with Hamiltonian Monte Carlo (HMC). HMC sampling provides a substantial gain in efficiency over the common Metropolis-Hastings-based Markov Chain Monte Carlo, which currently is the most common approach for Bayesian tree estimation.

Our method PhyloGrad efficiently calculates the gradient of the log likelihood with respect to all elements of *Q*, including with respect to independent per-column substitution matrices, which is inaccessible with current phylogenetic software. This allows for very flexible and efficient methods of fitting these substitution matrices. It is easy to add regularization to prevent overfitting, and to use gradient-based optimization algorithms like LBFGS [9] or gradient decent.

First of all, we follow the reverse-mode differentiation approach, which has lower complexity than forward-mode differentiation for this task due to the possibly high number of model parameters. This idea builds on results of Ji et al. [10], who show how to compute the gradient with respect to branch lengths in linear instead of quadratic time with respect to the number of sequences.

The implementation of PhyloGrad is highly parallel and memory-efficient. We simplify the computation graph of Felsenstein’s algorithm, reducing the overhead of reverse-mode differentiation. All time critical computations are vectorized and optimized for memory locality.

In addition to these engineering improvements, a key step is optimizing the differentiation of the matrix exponential. The work of McGibbon and Pande [11] describes an approach to obtain an efficient and numerically stable solution for the forward-mode differential. We derive how to apply this approach also in the reverse-mode setting. Motivating and describing this computation is the main topic of the present article.

## Implementation

### From rate matrix to likelihood

In a CTMC model, the rate matrix *Q*, sometimes also called the generator matrix, specifies the infinitesimal rate of transition for each pair of residues. The transition probabilities over any specific time *t* can then be obtained from this matrix: namely, the probability of transitioning from residue *a* to residue *b* in time *t* is given by $(e^{Q t})_{a b}$. The rate matrix has non-negative off-diagonal entries, and each of its rows sums up to 0. In particular, the diagonal elements will be negative.

Felsenstein’s Algorithm [1] uses transition probabilities to calculate the log likelihood of observing the sequences of an MSA given a phylogentic tree. For each combination of a substitution matrix and an branch length in the tree we need to invoke one matrix exponential to get corresponding transition probabilities. When there are different rate matrices for different columns, one cannot reuse the transition probabilities and incurs a large computational cost.

Implementing this algorithm directly in Pytorch can calculate the desired gradients using automatic differentiation, but most of the time is spent in calculating the matrix exponential and its gradient.

### Replacing the matrix exponential

The matrix exponential is a function from $\mathbb {R}^{n \times n} \rightarrow \mathbb {R}^{n \times n}$ with $e^X:= \sum _{i = 0}^\infty \frac{X^i}{i!}$. Evaluating $e^X$ for an arbitrary input matrix *X* is quite expensive, as even the best available algorithms involve a significant number of matrix multiplications in this case [12]. We provide timings for random inputs in Appendix C.

If the matrix *Q* is diagonalized, $Q = A \Lambda A^{-1}$ with a diagonal matrix $\Lambda = {{\,\textrm{diag}\,}}(\lambda _1,...,\lambda _n)$ and an invertible matrix $A \in \mathbb {R}^{n \times n}$, we can calculate the matrix exponential $e^{Q t} = A e^{\Lambda t} A^{-1}$ for time $t>0$ using only *n* scalar $\exp (\lambda _i t)$ evaluations and two matrix multiplications. Notice that, although the computational cost of diagonalization is comparable to that of matrix exponentiation, we only have to compute it once for a given rate matrix *Q* and can then reuse it for different values of *t*. Because in Felsenstein’s algorithm we have as many different branch lengths *t* as we have branches in the phylogenic tree, diagonalization gives a significant speedup compared to the direct computations of each of the matrix exponentials.

Unfortunately, diagonalizing the matrix makes the gradient calculation numerically unstable, because the gradient of eigenvectors with respect to a matrix gets unstable when two eigenvalues get close to each other and is even undefined for repeated eigenvalues [13].

In the following we present a strategy to apply the diagonalization optimization and still get numerically stable gradients.

### Forward- and reverse-mode differentiation

To better motivate our algorithm, we give here a short review on automatic differentiation.

Suppose our model is parameterized by a vector $\theta \in \mathbb {R}^{m}$. So instead of $Q \in \mathbb {R}^{n \times n}$, we have a function $Q(\theta )$. For the most general time-reversible model, $\theta$ would have $m = n(n-1)/2$ dimensions (see section about time symmetric parametrization).

For calculation of gradients, it seems natural to calculate the gradient with respect to each parameter $\theta _u$ of all the intermediate expressions in the algorithm and eventually the final log likelihood. This approach is called forward-mode differentiation, because the gradients are calculated in the same order as the values.

This turns out to be inefficient because you will have to calculate $\frac{ \partial e^{Qt}}{\partial \theta _u}$ for all the different times *t*. For each of the *m* parameters in $\theta$, there are $n^2$ partial derivatives, so $n^2m$ in total. Evaluating the $n^2$ partial derivatives with respect to one parameter will take $\mathcal {O}(n^3)$ operations. Overall, this will lead to a complexity of $\mathcal {O}(n^3m)$, which will be $\mathcal {O}(n^5)$ in the case of $m = n(n-1)/2$ parameters.

Reverse-mode differentiation is a strategy for calculating gradients of any function. In case of scalar output, it will have the same complexity as evaluating the function. The idea is to first calculate the log likelihood *L*. Then you work back and calculate the derivative of *L* with respect to the intermediate expressions in the algorithm and eventually with respect to the parameters. So for example instead of $\frac{ \partial e^{Qt}}{\partial \theta _u}$ we calculate $\frac{\partial L}{\partial e^{Qt}}$. So we reduce the number of partial derivatives from $n^2m$ to $n^2$. The drawback is that we have to calculate the log likelihood *L* first and save the necessary information which is needed to perform the backward pass.

In general, forward-mode differentiation is more efficient if you have lower parameter dimension than output dimension, while reverse-mode differentiation is more efficient if you have higher parameter dimension than output dimension. This is always the case for a scalar function.

There are a lot of frameworks that automate this task, such as Pytorch, Tensorflow and Jax. The main problem is that it is not clear how to apply the diagonalization optimization to save the matrix exponential calculation, and a direct implementation leads to the above-discussed numerical instabilities. Moreover, automatic differentiation libraries typically incur some overhead compared to a well-designed manual implementation. The amount can vary a lot depending on the algorithm and framework. In our case with Pytorch we see an immensely higher memory consumption than in our manual implementation, see Fig. 2.

### Numerically stable matrix exponential gradients

Reference [14] gives in Theorem 4.5 a useful formula for the case with a diagonalization $Q = A \Lambda A^{-1}$ (see our proof in Appendix A):

1 $$\begin{aligned} \frac{ \partial e^{Qt}}{\partial \theta _u} = A \left( A^{-1} \frac{ \partial Q}{\partial \theta _u} A \odot X(\Lambda , t) \right) A^{-1} \end{aligned}$$

with

$$ X(\Lambda , t)_{i j} := {\left\{ \begin{array}{ll} t \exp (t \lambda _i) & \text {if } i = j \\ \frac{\exp (t \lambda _i) - \exp (t \lambda _j)}{\lambda _i - \lambda _j} & \text {if } j \ne i , \end{array}\right. } $$

and $\odot$ denoting the element-wise multiplication (Hadamard product). The matrix $X(\Lambda , t)$ can be computed in a numerically stable way even when the eigenvalues are close to each other or identical.

However, this formula is only applicable in the forward-mode differentiation setting, where we first calculate $\frac{ \partial Q}{\partial \theta _u}$ and then $\frac{ \partial e^{Qt}}{\partial \theta _u}$. For reverse-mode differentiation we need to go from $\frac{ \partial L}{\partial e^{Qt}}$ to $\frac{ \partial L}{\partial Q}$.

### Going from forward- to reverse-mode differentiation

Lets look at a simplified setting of calculating $L(\theta )$:

$$\begin{aligned} \theta \rightarrow Q \rightarrow e^{Qt} \rightarrow L\Big ( e^{Q(\theta ) t}\Big ) \end{aligned}$$

Here *t* is constant, and we are interested in the gradient of the log likelihood function *L* with respect to the parameters $\theta$ of matrix $Q(\theta )$. To make things simpler we will think about *Q* as an $\mathbb {R}^{n^2}$ vector by flattening it. The Jacobian Matrix $\frac{\partial e^{Qt}}{\partial Q}$ has $n^2 \times n^2$ entries and represents a linear mapping from $\mathbb {R}^{n ^ 2}$ to $\mathbb {R}^{n ^ 2}$. By the chain rule we know that

2 $$\begin{aligned} \frac{ \partial e^{Qt}}{\partial \theta _u} = \frac{\partial e^{Qt}}{\partial Q} \, \frac{ \partial Q}{\partial \theta _u} \end{aligned}$$

and also

3 $$\begin{aligned} \frac{\partial L}{\partial Q} = \frac{\partial L }{\partial e^{Qt}} \, \frac{\partial e^{Qt}}{\partial Q} \end{aligned}$$

Equation 2 is a matrix–vector multiplication and Eq. 3 is a vector–matrix multiplication. So we can think about the Jacobian as a linear transformation, $\frac{\partial e^{Qt}}{\partial Q}:\frac{ \partial Q}{\partial \theta _u} \mapsto \frac{ \partial e^{Qt}}{\partial \theta _u}$, and the transposed Jacobian as $\left( \frac{\partial e^{Qt}}{\partial Q}\right) ^{\!\!\top }: \frac{\partial L }{\partial e^{Qt}} \mapsto \frac{\partial L}{\partial Q}$.

Equation 1 allows to evaluate Eq. 2 without computing all of the $n^4$ entries of the Jacobian. For reverse-mode differentiation, we need to evaluate Eq. 3 efficiently. So we need to find the corresponding expression for the transposed Jacobian.

We now consider the vector space of (non flattened) $n \times n$ matrices. We define the standard scalar product $\langle A, B \rangle := \sum _{i,j} A_{i j} B_{i j} = \sum _j (\sum _{i} (A^\top )_{ji} B_{i j}) ={{\,\textrm{Tr}\,}}(A^\top B)$. The Jacobian is a linear map from this space to itself.

In general for any linear map $F: \mathbb {R}^{n\times n} \rightarrow \mathbb {R}^{n\times n}$ and any pair of matrices *N*, *M* we have $\langle F(N),M \rangle = \langle N, F^\top (M) \rangle$, where $F^\top$ is the transposed map. The reverse is also true, that a map $F^\top$ which has this property must the transposed map. We use $A^{-\top }$ to denote the inverse of the transposed matrix.

Let’s take two arbitrary $n \times n$ matrices *M* and *N*:

4 $$\begin{aligned}&\langle A (A^{-1} M A \odot X(\Lambda , t))A^{-1}, N \rangle = \nonumber \\ \text {(by def. of the scalar product)} =&{{\,\textrm{Tr}\,}}(A^{-\top } (A^{-1} M A \odot X(\Lambda , t))^\top A^\top N) \nonumber \\ \text {(by cyclic permutation in trace)} =&{{\,\textrm{Tr}\,}}((A^{-1} M A \odot X(\Lambda , t))^\top A^\top N A^{-\top }) \nonumber \\ \text {(by lemma in appendix B)}=&{{\,\textrm{Tr}\,}}( A^\top M^\top A^{-\top } (A^\top N A^{-\top } \odot X(\Lambda , t)) ) \nonumber \\ \text {(by cyclic permutation in trace)} =&{{\,\textrm{Tr}\,}}(M^\top A^{-\top } (A^\top N A^{-\top } \odot X(\Lambda , t)) A^\top ) \nonumber \\ \text {(by def. of the scalar product)} =&\langle M ,A^{-\top } (A^\top N A^{-\top } \odot X(\Lambda , t)) A^\top \rangle \end{aligned}$$

So, the linear map $N \rightarrow A^{-\top } (A^\top N A^{-\top } \odot X(\Lambda , t)) A^\top$ is the transposed Jacobian and we can use it in the backward pass of reverse-mode differentiation.

After the diagonalization of $Q = A \Lambda A^{-1}$, we can compute

5 $$\begin{aligned} \frac{\partial L}{\partial Q} = A^{-\top } \big (A^\top \frac{\partial L}{\partial e^{Qt}} A^{-\top } \odot X(\Lambda , t) \big ) A^\top \end{aligned}$$

with just four matrix multiplications for arbitrary *t*. In the Felstenstein algorithm we sum up all the $\frac{\partial L}{\partial Q}$ contributions over all the branches, so we can factor out the two outermost matrix multiplications.

### Parametrization for time-reversible models

The vast majority of phylogenetic models assume a condition called time reversibility. As shown in [11], this condition allows one to choose a convenient parametrization for the rate matrix, and ensures that it can be diagonalized, as summarized below.

For a vector $\pi$, we denote by $\sqrt{\pi }$ its component-wise square root, and by ${{\,\textrm{diag}\,}}(\pi )$ the diagonal matrix whose entries are specified by $\pi$.

A CTMC model is called time-reversible if there exists a distribution $\pi$ over its states such that $\pi _i q_{i j} = \pi _j q_{j i}$ for all *i*, *j*, or, equivalently, ${{\,\textrm{diag}\,}}(\pi )Q = Q^\top {{\,\textrm{diag}\,}}(\pi )$. By transforming the expression into ${{\,\textrm{diag}\,}}(\sqrt{\pi }) Q {{\,\textrm{diag}\,}}(\sqrt{\pi })^{-1} = {{\,\textrm{diag}\,}}(\sqrt{\pi })^{-1} Q^\top {{\,\textrm{diag}\,}}(\sqrt{\pi })$, we see that $S:={{\,\textrm{diag}\,}}(\sqrt{\pi }) Q {{\,\textrm{diag}\,}}(\sqrt{\pi })^{-1}$ is a symmetric matrix.

First of all, this implies that *Q* can be parametrized in terms of the symmetric matrix *S* and the vector $\pi$ via $Q = {{\,\textrm{diag}\,}}(\sqrt{\pi })^{-1} S {{\,\textrm{diag}\,}}(\sqrt{\pi })$. By definition, *Q* must have non-negative off-diagonal elements, with diagonal entries chosen so that each row sums to 0. Thus only the upper triangular entries of *S* need to be explicitly specified in the parametrization.

In addition, recall that for an arbitrary matrix, a diagonalization may not exist, or it may be numerically unstable to compute. On the other hand, symmetric matrices are always diagonalizable in an efficient and numerically stable way, their eigenvectors are orthogonal and their eigenvalues are real numbers.

*S* is symmetric, and therefore has a diagonalization *S* =* B* Λ * B*^-1^ with * B*^-1^ = * B*^T^. Substituting back, we get $Q = {{\,\textrm{diag}\,}}(\sqrt{\pi })^{-1}B \Lambda B^{-1} {{\,\textrm{diag}\,}}(\sqrt{\pi })$. This gives a diagonalization for *Q* as $Q = A \Lambda A^{-1}$ with $A:= {{\,\textrm{diag}\,}}(\sqrt{\pi })^{-1} B$ and $A^{-1} = B^{-1}{{\,\textrm{diag}\,}}(\sqrt{\pi }) = B^\top {{\,\textrm{diag}\,}}(\sqrt{\pi })$. These expressions are numerically stable as long as no entry of $\sqrt{\pi }$ is too small. The implementation treats every value of $\sqrt{\pi } < 10^{-10}$ as $10^{-10}$ to avoid instabilities.

The optimization described in the previous two sections works whenever *Q* can be diagonalized, regardless of time-reversibility. Still, we assume that *Q* is time-reversible in PhyloGrad. It is a commonly satisfied condition, and it lets us provide a numerically stable parametrization for *Q* for the user’s convenience.

IQ-TREE and RaxML use the parametrization $Q = R {{\,\textrm{diag}\,}}(\pi )$, where *R* is a symmetric matrix. Parametrizing in terms of $\sqrt{\pi }$ instead of $\pi$ makes our approach slightly more numerically stable.

### Code architecture

We have implemented reverse-mode differentiation for all parts of Felsenstein’s algorithm in the Rust programming language. We use our specialized method for the matrix exponential and standard approaches for all the other operations.

We provide Python bindings that take as input a phylogenetic tree and the residue profiles associated to its leaves (in particular, it can accept one-hot-encoded residue sequences, but more general profiles that account for sequencing ambiguity are also allowed). Given an equilibrium distribution $\sqrt{\pi }$ and an upper triangular matrix *S*, the package calculates the gradients of the log likelihood with respect to these two inputs.

The implementation parallelizes over multiple cores with the help of the Rust crate rayon, which is similar to OpenMP in the C/C++ world. As an alternative, we also provide a Jax implementation, which is able to also run on a GPU or TPU. On CPU, it is not as efficient as our Rust implementation.

## Results

### Comparison to IQ-TREE and RaxML

We want to compare the optimization of a global *Q* matrix between PhyloGrad and the IQ-TREE implementation provided by QMaker [15] and RaxML [3]. This case is computationally easier than the more general case of an independent rate matrix for each column. For better performance, PhyloGrad has an extra code path for the special case of a single, global matrix.

All three methods are gradient-based and are using LBFGS [16]. QMaker and RaxML use (one-sided) finite differences to obtain the gradients, which needs 209 Felsenstein evaluations for one gradient update (208 free parameters to optimize and 1 evaluation for the other term in the finite difference).

Our algorithm needs only one forward and one backward pass through the Felsenstein algorithm. Both passes are more complicated and therefore time-intensive. We calculate the partial likelihoods in log-space, which is simpler but slower than the optimized implementations in IQ-TREE and RaxML.

The trees are randomly generated and the alignments are simulated with IQ-TREE using the LG model. The results can be seen in Table 3 for single-threaded performance and Table 2 for multi-threaded performance.

We see that RaxML is considerably slower than IQ-TREE, but also produces better likelihoods, see Apendix D. We optimize until one LBFGS iteration does not improve likelihood by more than 0.0001%, which puts us close to the likelihood of RaxML and quite a bit ahead of IQ-TREE.

We can see a consistent speedup of a factor 10 while obtaining a better likelihood than IQ-TREE. In the multi-threaded run the difference is even larger.

We ran and compiled on a machine with two AMD EPYC 7742 64-Core Processors, making sure only one task was running on each machine at a time. The exact benchmark code can be found in the repository. Log files can be found in the logs.zip supplementary file. Table 1

If we define a partition for each column, we can make IQ-TREE and RaxML optimize a column-specific GTR model. Of course, this is extremely over-parametrized, but the gradients with respect to the full GTR model can be used to train very general but lower dimensional models, see for example our Pytorch benchmark. Table 2

IQ-TREE and RaxML now have to do a matrix diagonalization for each of the 209 Felsenstein passes per column. We only need to diagonalize once per column. There might also be considerable overhead in IQ-TREE and RaxML because they are not designed for single column partitions. Table 4

IQ-TREE does not seem to work properly in this setting and stops optimization prematurely. You can see this in the final likelihoods in the Appendix E. RaxML optimizes to very similar values than our method, but takes 100 to 1000 times as long. These extreme differences are probably due to poor optimization of this rather special use-case in RaxML.

**Table 1:** Benchmarking of global GTR model of IQ-TREE and RaxML vs PhyloGrad with 1 thread

Tree size	Alignment size	IQ-TREE (s)	RaxML (s)	PhyloGrad (s)
16	50	9 / 13.0x	22 / 32.5x	0.68
64	50	29 / 9.6x	91 / 30.1x	3.04
256	50	76 / 7.8x	409 / 42.0x	9.73
1024	50	388 / 11.5x	2669 / 79.1x	33.76
4096	50	2762 / 28.9x	10094 / 105.8x	95.44
16	200	16 / 9.0x	55 / 31.9x	1.74
64	200	106 / 10.9x	224 / 23.0x	9.74
256	200	297 / 9.6x	1201 / 38.7x	31.03
1024	200	1107 / 9.9x	5304 / 47.2x	112.47
4096	200	6002 / 14.0x	12649 / 29.5x	428.72

**Table 2:** Benchmarking of global GTR model of IQ-TREE and RaxML vs PhyloGrad with 32 threads

Tree size	Alignment size	IQ-TREE (s)	RaxML (s)	PhyloGrad (s)
16	50	6 / 25.6x	28 / 128.1x	0.22
64	50	15 / 23.0x	120 / 178.4x	0.67
256	50	126 / 74.4x	542 / 319.1x	1.70
1024	50	170 / 27.7x	4518 / 737.0x	6.13
4096	50	1296 / 82.9x	16779 / 1073.5x	15.63
16	200	9 / 35.5x	32 / 122.4x	0.26
64	200	22 / 18.9x	108 / 93.2x	1.16
256	200	74 / 22.2x	686 / 204.8x	3.35
1024	200	226 / 14.1x	4536 / 281.9x	16.09
4096	200	1356 / 25.2x	10832 / 201.1x	53.86

**Table 3:** Benchmarking of the per column GTR model of IQ-TREE and RaxML vs PhyloGrad with 1 thread. IQ-TREE stops optimizing very early, so these times are not representative

Tree size	Alignment size	IQ-TREE (s)	RaxML (s)	PhyloGrad (s)
16	10	0.45 / 1.7x	46.0 / 170x	0.27
64	10	2.07 / 0.9x	2145 / 887x	2.42
256	10	656.44 / 49.4x	9931 / 747x	13.30
16	50	4.28 / 2.7x	392.9 / 246x	1.60
64	50	15.75 / 1.3x	16858 / 1350x	12.49
256	50	3973.0 / 60.7x	76325 / 1165x	65.50

**Table 4:** Benchmarking of IQ-TREE and RaxML vs PhyloGrad with 5 threads. IQ-TREE does not run with 10 columns and 5 threads

Tree size	Alignment size	IQ-TREE (s)	RaxML (s)	PhyloGrad (s)
16	10	-	15.6 / 194.4x	0.08
64	10	-	733.1 / 1078.1x	0.68
256	10	-	3369.3 / 893.7x	3.77
16	50	1.41 / 3.1x	132.3 / 287.6x	0.46
64	50	5.06 / 1.5x	5486.0 / 1672.6x	3.28
256	50	1246 / 69.1x	25168.0 / 1394.3x	18.05

### Comparison to Pytorch

We also want to compare our implementation against another reverse differentiation approach. We could not find a already implemented software for our specific task of column-specific gradients, so we compare PhyloGrad to our own Pytorch implementation of Felsenstein’s algorithm. The Pytorch implementation has the same asymptotic time complexity, because it is also based on reverse-mode differentiation, but it does not take advantage of the matrix exponential optimization and has the potential overhead of automatic differentiation.

For benchmarking, we choose a simple model which has a shared *S* for all columns but a column specific $\pi$. This is similar to the Cxx models [6] but instead of predefined categories for the $\pi$ we can learn a column specific $\pi$.

When there is not enough data to fit so many parameters without over-fitting, it is easy to parametrize $\pi$ with something lower-dimensional and learn an embedding which is shared for all columns. We think that this flexibility will be key for advancing more flexible phylogenetic models in the future.

We generated 300 random columns with different numbers of taxa in the tree and gave the program 64 threads. CPU versions are executed on a machine with 2 AMD EPYC 7742 64-Core Processors and 1TiB memory. GPU versions are executed on a machine with 2 AMD EPYC 7513 32-Core Processors and a A100 80GB GPU.

**Fig. 1:**
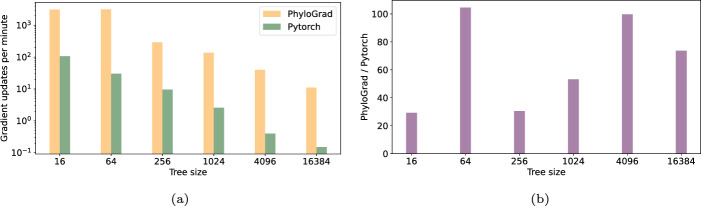
**a** Number of iterations per minute with 300 columns on 64 threads on CPU. **b** Relative speedup of PhyloGrad vs our Pytorch implementation of Felsenstein with 300 columns on 64 threads on CPU

We measure how many gradient updates we can do in 20 min and normalize to iterations per minute. Figure 1 shows the gradient updates per minutes for the Pytorch implementation and PhyloGrad. Depending on the tree size PhyloGrad is 30-100 times faster. Figure 3 shows the same but for GPU. Here we are 10-30 times faster.

Finally, we compare memory usage on CPU, where PhyloGrad is dramatically more memory-efficient.

**Fig. 2:**
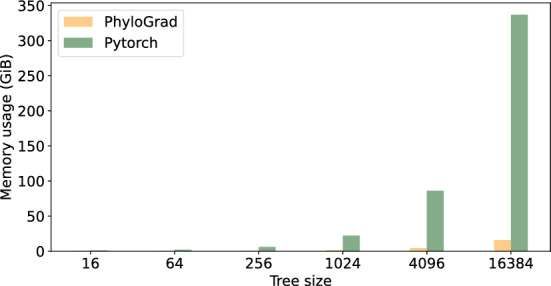
Memory consumption on the CPU with 64 threads on 300 columns

**Fig. 3:**
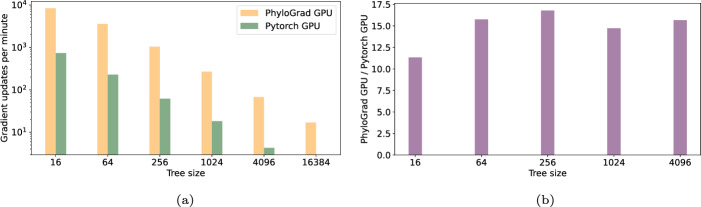
**a** Number of iterations per minute with 300 columns with 64 CPU threads on GPU. For the biggest tree size, Pytorch crashes because it needs more than 80GiB of memory. **b** Relative speedup of PhyloGrad vs our Pytorch implementation of Felsenstein with 300 columns with 64 CPU threads on GPU

## Conclusion

Our algorithm and implementation is significantly faster than simple reverse-mode differentiation in Pytorch and the current IQ-TREE and RaxML version. It can be combined with automatic differentiation frameworks, allowing easy definition and efficient fitting of any time-reversible model. The considerable speed-up provided by PhyloGrad will enable the learning of evolutionary models with many more parameters than currently possible. We expect that PhyloGrad will thereby facilitate the development of more realistic and accurate phylogenetic tree reconstruction and ancestral sequence reconstruction methods.

A preprint [17] by Ji et al., published shortly before the submission of this paper, also offers fast gradients of the phylogenetic model likelihood. The article deals with the optimization of branch-specific parameters, while our focus is on column-specific ones, so the implementations cannot directly be compared. Their approach is based on [10] and therefore can leverage already well implemented Felsenstein implementations. Our current implementation uses the logsumexp function for numerical stability which could be improved. On the other hand, in the algorithm of Ji et al. one has to re-evaluate the equation (11) from [17], for every parameter that requires gradients, so it can probably be improved using the ideas from this article. A combination of both ideas will probably yield the best results for phylogenetic gradients in the column and the branch specific case.

## Availability and requirements

**Project name:** PhyloGrad

**Project home page:** https://github.com/soedinglab/phylo_grad

**Operating system:** Platform independent, tested on Linux

**Programming language:** Rust and Python

**Other requirements:** Python 3.7 or newer, Rust compiler to compile from source

**License:** MIT or Apache2

**Any restrictions to use by non-academics:** No

## Supplementary Information

Supplementary file 1 (zip 4800 KB)

## Figures and Tables

**Fig. 4:**
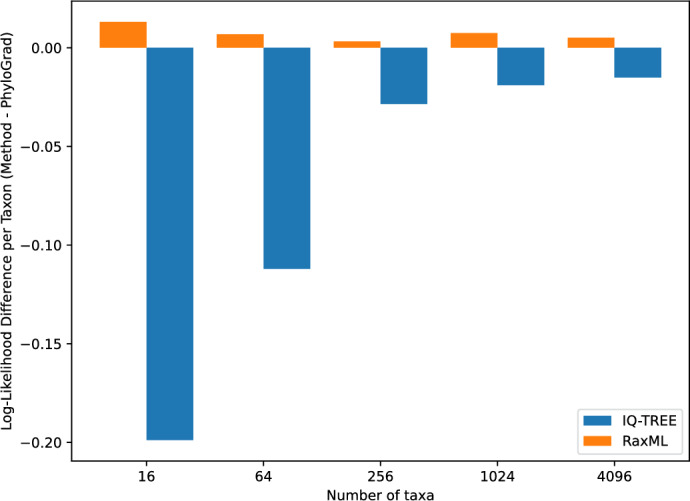
Difference in log likelihood divided by number of taxa on 200 columns

**Fig. 5:**
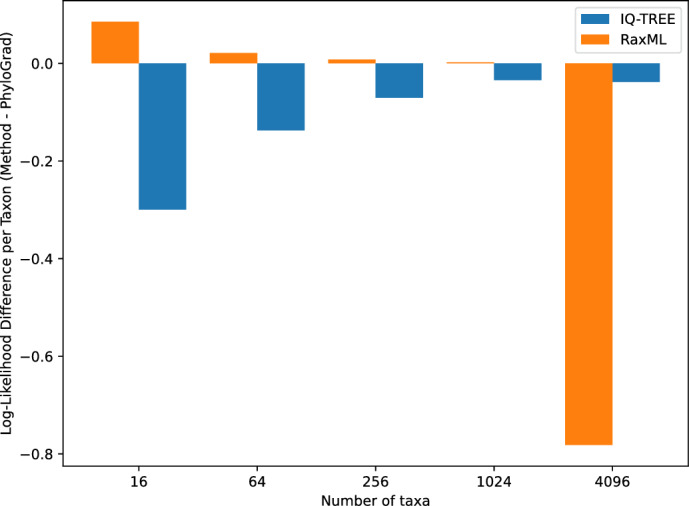
Difference in log likelihood divided by number of taxa on 200 columns. RaxML fails to optimize on the largest tree

**Fig. 6:**
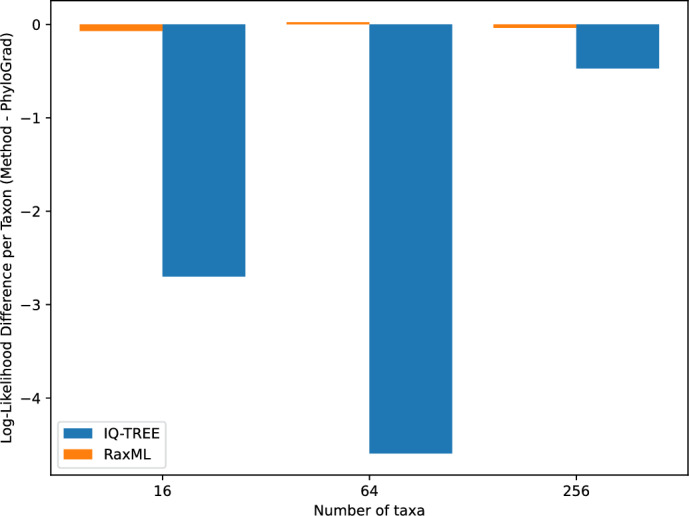
Difference in log likelihood divided by number of taxa on 10 columns

**Fig. 7:**
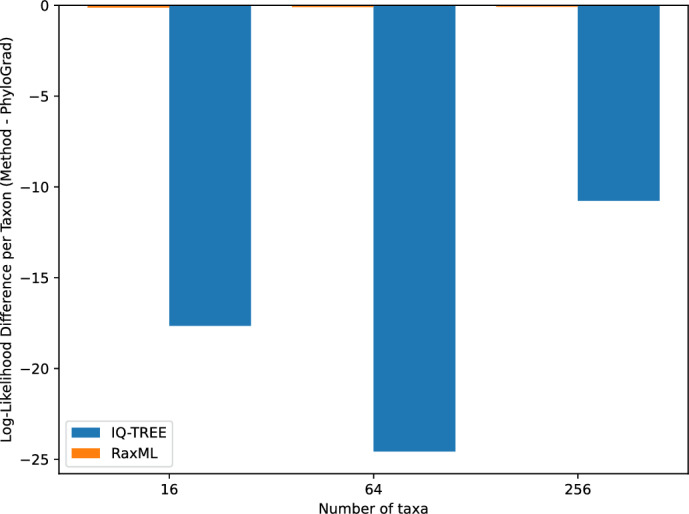
Difference in log likelihood divided by number of taxa on 50 columns

## Data Availability

We use only synthetic data for the benchmark. The generation process can be reproduced with the snakemake pipeline in the github repository. The actual files used for our benchmark can also be found in the repository.

## Appendix A Proof of equation (1)

Application of the definition of matrix exponential gives $e^{Qt} = \sum _{k=0}^\infty \frac{(A \Lambda A^{-1} \,t)^k}{k!} = A \left( \sum _{k=0}^\infty \frac{(\Lambda t)^k}{k!} \right) A^{-1} = A e^{\Lambda t} A^{-1}$. We will also need

A1 $$\begin{aligned} \frac{\partial A^{-1}}{\partial \theta _u} = - A^{-1} \frac{\partial A}{\partial \theta _u} A^{-1} \end{aligned}$$

which follows from $0 = \frac{\partial (A^{-1} A)}{\partial \theta _u} = \frac{\partial A^{-1}}{\partial \theta _u} A + A^{-1} \frac{\partial A}{\partial \theta _u}$. We abbreviate $D:= A^{-1} \frac{\partial A}{\partial \theta _u}$ to obtain

A2 $$\begin{aligned} A^{-1} \frac{\partial e^{Qt}}{\partial \theta _u} A&= A^{-1} \left( \frac{\partial A}{\partial \theta _u} e^{\Lambda t} A^{-1} + A \frac{\partial e^{\Lambda t}}{\partial \theta _u} A^{-1} + A e^{\Lambda t} \Big ( - A^{-1} \frac{\partial A}{\partial \theta _u} A^{-1} \Big ) \right) A \nonumber \\&= D e^{\Lambda t} + \frac{\partial e^{\Lambda t}}{\partial \theta _u} - e^{\Lambda t} D . \end{aligned}$$

The matrix elements for $i \ne j$ of the sum of the first and third term are

A3 $$\begin{aligned} \left( D e^{\Lambda t} - e^{\Lambda t} D \right) _{ij}&= D_{ij} e^{\lambda _j t} - e^{\lambda _i t} D_{ij} = \big ( D_{ij} \lambda _j - \lambda _i D_{ij} \big ) \frac{e^{\lambda _i t} - e^{\lambda _j t}}{\lambda _i - \lambda _j} \nonumber \\&= \big ( (D \Lambda - \Lambda D) \odot X(\Lambda ,t) \big )_{ij}\,. \end{aligned}$$

For $i=j$, equation (A3) still holds as both sides vanish. The second term in the equation (A2) is

A4 $$\begin{aligned} \left( \frac{\partial e^{\Lambda t}}{\partial \theta _u} \right) _{ij}&= \frac{\partial e^{\lambda _i t}}{\partial \theta _u} \, \delta _{ij} = \frac{\partial \lambda _i}{\partial \theta _u} t \, e^{\lambda _i t} \, \delta _{ij} = \left( \frac{\partial \Lambda }{\partial \theta _u} \odot X(\Lambda , t) \right) _{ij}\,. \end{aligned}$$

Using the equations (A2), (A3), and (A4), we obtain

A5 $$\begin{aligned} A^{-1} \frac{\partial e^{Qt}}{\partial \theta _u} A&= (D \Lambda - \Lambda D) \odot X(\Lambda ,t) + \frac{\partial \Lambda }{\partial \theta _u} \odot X(\Lambda , t) \nonumber \\&= \left( A^{-1} \frac{\partial A}{\partial \theta _u} \Lambda + \frac{\partial \Lambda }{\partial \theta _u} - \Lambda A^{-1} \frac{\partial A}{\partial \theta _u} \right) \odot X(\Lambda ,t) \nonumber \\&= \left( A^{-1} \frac{\partial A}{\partial \theta _u} \Lambda + \frac{\partial \Lambda }{\partial \theta _u} + \Lambda \frac{\partial A^{-1}}{\partial \theta _u} A \right) \odot X(\Lambda ,t) \nonumber \\&= \left( A^{-1} \frac{\partial }{\partial \theta _u}\left( A \Lambda A^{-1} \right) A \right) \odot X(\Lambda ,t), \end{aligned}$$

which concludes the proof.

## Appendix B Hadamard Product Lemma

If $A, B, \Lambda \in \mathbb {R}^{n \times n}$ are three square matrices, then ${{\,\textrm{Tr}\,}}((A \odot \Lambda )^{\top } B) = {{\,\textrm{Tr}\,}}(A^\top (B \odot \Lambda ))$.

Proof: By definition of the trace and the matrix product, the left hand side is

$$ \sum _{i = 1}^n \sum _{k = 1}^n (A_{ki} \Lambda _{ki}) B_{ki} .$$

Using the same definitions to expand the right hand side, we get

$$ \sum _{i = 1}^n \sum _{k = 1}^n A_{ki} (B_{ki} \Lambda _{ki}),$$

equal to the previous expression.

## Appendix C Matrix Exp Performance

We tested with pytorch 2.5.1 on a an Intel i7-11700K using the jupyter lab timeit command.

## Appendix D Likelihood of global matrix benchmark

See Figs. 4 and 5.

## Appendix E Likelihood of matrix per column benchmark

See Figs. 6 and 7

## Abbreviations

MSA: Multiple Sequence Alignment
CTMC: Continuous Time Markov Chain
MCMC: Markov Chain Monte Carlo
HMC: Hamiltonian Monte Carlo
